# How accurate can genetic predictions be?

**DOI:** 10.1186/1471-2164-13-340

**Published:** 2012-07-24

**Authors:** Jonathan M Dreyfuss, Daniel Levner, James E Galagan, George M Church, Marco F Ramoni

**Affiliations:** 1 Partners Center for Personalized Genetic Medicine, Boston, MA, 02115, USA; 2 Graduate Program in Bioinformatics, Boston University, Boston, MA, 02215, USA; 3 Department of Genetics, Harvard Medical School, Boston, MA, 02115, USA; 4 Wyss Institute for Biologically Inspired Engineering, Harvard University, Boston, MA, 02115, USA; 5 Broad Institute of MIT and Harvard, Cambridge, MA, 02142, USA; 6 Department of Biomedical Engineering, Boston University, Boston, MA, 02215, USA; 7 Harvard-MIT Division of Health Sciences and Technology, Boston, MA, 02139, USA; 8 Children’s Hospital Informatics Program, Boston, MA, 02115, USA

## Abstract

**Background:**

Pre-symptomatic prediction of disease and drug response based on genetic testing is a critical component of personalized medicine. Previous work has demonstrated that the predictive capacity of genetic testing is constrained by the heritability and prevalence of the tested trait, although these constraints have only been approximated under the assumption of a normally distributed genetic risk distribution.

**Results:**

Here, we mathematically derive the absolute limits that these factors impose on test accuracy in the absence of any distributional assumptions on risk. We present these limits in terms of the best-case receiver-operating characteristic (ROC) curve, consisting of the best-case test sensitivities and specificities, and the AUC (area under the curve) measure of accuracy. We apply our method to genetic prediction of type 2 diabetes and breast cancer, and we additionally show the best possible accuracy that can be obtained from integrated predictors, which can incorporate non-genetic features.

**Conclusion:**

Knowledge of such limits is valuable in understanding the implications of genetic testing even before additional associations are identified.

## Background

Accurate pre-symptomatic prediction of disease and drug response is a vital component of personalized medicine, which could allow for improved clinical decision-making and targeted prevention strategies, easing both the burden and costs of disease [1]. Already, several companies offer consumers personalized risk assessments, lifestyle recommendations, and 'nutraceuticals' based on their genetic profiles [2]. Unfortunately, most genetic factors associated with common traits explain only a small portion of the phenotypic variance (the “missing heritability” problem [3]), making genetic prediction currently difficult [4]. Investment into studies that assay rare variants [5] and the use of informative polymorphisms that do not individually pass stringent statistical tests of association [6] can improve the accuracy of predictions, but the extent to which predictions can be improved is unclear. Thus, identifying the bounds on the accuracy of predictive genetic testing based on readily-known disease parameters (such as prevalence and heritability) can be an invaluable planning tool.

Although the accuracy of a medical test can be measured in many ways, the concepts of sensitivity and specificity are paramount [7]. Frequently, the test result is continuous (e.g. the individual’s predicted risk), while the clinical decision and true outcome are binary (e.g. either the person will get sick or not), so that different thresholds of the test result yield different pairs of sensitivity and specificity. The receiver operator characteristic (ROC) curve depicts this tradeoff between sensitivity and specificity across all possible thresholds, and the area under this curve (AUC) is the most widely used metric to summarize the accuracy of a test. An AUC of 1 indicates perfect prediction while an AUC of 0.5 represents random guessing.

Evidence that a bound on maximum predictive accuracy exists can be found in heritability. The heritability of a trait (in the broad-sense) is the proportion of phenotypic variation in the population that can be attributed to genetic variation; that is, it reflects the contribution of genetic factors relative to environmental ones. Narrow-sense heritability measures the corresponding quantity for additive genetic variance only, which excludes genetic effects such as dominance and epistasis. The heritability of binary phenotypes can be computed directly on the observed binary scale. However, it may also be calculated on a liability scale, where it is assumed that an individual has the binary trait if their risk exceeds a threshold. Both types of heritability can be estimated using family-based studies, such as twin studies [8], and the two scales can be mapped to each other [9].

The impact of heritability on genetic test accuracy can be seen by examining its two extremes: a trait that has 100% heritability, such as a Mendelian trait, can be predicted with certainty from the genotype; in contrast, a trait with 0% heritability is not influenced by genetic factors, and thus genetic tests cannot produce any useful information. Previous ground-breaking works have investigated the bounds prevalence and heritability impose on predictive accuracy using simulations [10], analytical results utilizing genotype relative risks and their frequencies [11], and analytical approximations under the assumption of a normally distributed liability [12,13]. Here, we mathematically elucidate the absolute bounds on the specificities, sensitivities, and AUC for genetic testing given any values of heritability and prevalence of the tested trait, without making any assumptions about the risk distribution.

## Results

Common complex traits are typically the combined effect of genetic and environmental factors. Since no practical predictor can account for all factors and their interactions, clinical prediction can at best assign probabilistic risks rather than deterministic outcomes. Viewed on the population level, these risk assignments can be seen as comprising a risk distribution, which is an estimate of the population’s true risk distribution. Maximal predictive accuracy occurs when the estimated risk matches the true risk.

The prevalence and heritability of any trait restrict the set of possible genetic risk distributions. If we know the risk corresponding to each individual’s genetic profile in a large sample, then we can obtain an expression for broad-sense heritability (H^2^) on the binary scale [10]:

(1) $$\text{heritability}=H^{2}=1-\frac{\sum_{i}risk_{i}\left(1-risk_{i}\right)}{\bar{risk}\left(1-\bar{risk}\right)n}$$

where *i* = *1,…,n* indexes people, *n* is the sample size, *risk*_*i*_ is individual *i*’s genetic risk (i.e. the conditional probability of the trait given genes), and $\bar{risk}$is the average genetic risk, which equals the average population risk (see Methods). The meaning of *risk* depends on the context: for instance, when the phenotype is current disease status, the average risk in the population is its prevalence, whereas in prediction of lifetime illness, *risk* is the lifetime risk of disease. (When possible, we nonetheless opt for the term *prevalence*.) Equation 1 mathematically expresses that *heritability* is the proportion of phenotypic variance explained by the genetic risk distribution.

**Figure 1:**
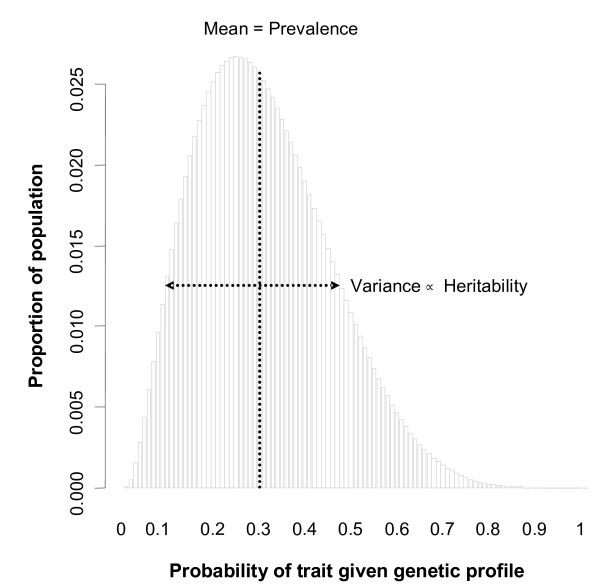
**Example risk distribution.** This distribution has a prevalence of 30% and a heritability of 10%. The mean of the distribution equals the prevalence of the trait. *Variance* represents the variance of risk due to genetic variation, sometimes called *genetic variance*, and is proportional to heritability.

To mathematically derive the risk distribution that yields the best genetic prediction, we model the distribution as a histogram with equally-spaced bins located from 0 to 100% representing risk groups, where the height of each bin denotes the proportion of the population who fall into that risk group (for an example, see Figure 1). This approach can define any risk distribution. Indeed, multiple genetic risk distributions can correspond to a given combination of prevalence and heritability; each such distribution, however, can lend itself differently to genetic prediction. Our method is based precisely on determining which such distribution (for a given prevalence and heritability) would allow the best predictive accuracy. Thus, for each combination of prevalence and heritability, we optimized the AUC that would be achieved if everyone’s risk were ideally ordered over the set of risk distributions that satisfied the combination of prevalence and heritability; similarly, we maximized the sensitivity for any given specificity, prevalence, and heritability over the set of risk distributions and thresholds that satisfied the constraints.

**Figure 2:**
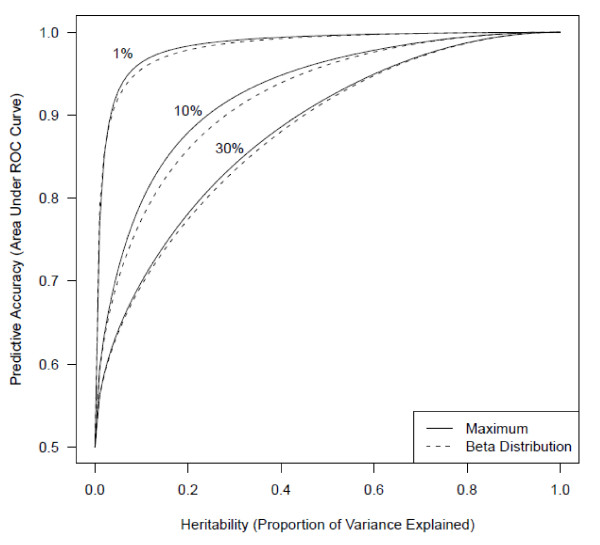
**Heritability vs. predictive accuracy.** Relationship of heritability (computed on the observed binary scale) or proportion of variance explained to the maximal upper limit on AUC. The numbers next to the curves represent the prevalence. The maximal AUCs are compared with those that would exist if the genetic risk distribution followed a beta distribution, which is consistent with previous reports [10,12,13].

Using this approach, we have derived the maximum limits on the genetic predictive accuracy of any binary trait given only its prevalence and heritability. These values are tabulated in Additional files 1 and 2 in terms of the AUC and sensitivity/specificity pairs, respectively. Additional file 3 contains computer code in the R software environment [14] for the algorithms we developed. Figure 2 displays AUC limits over all heritabilities for several prevalences, and it includes a comparison with the limits that would exist if genetic risk followed a beta distribution. The beta distribution is a flexible statistical distribution which is consistent with the assumptions of previous analytical approximations of the effect of prevalence and heritability on the ROC curve [12,13], because it can take the shape of countless smooth unimodal risk distributions. Furthermore, unlike previous approximations which deteriorate at high heritabilities [12], the beta distribution limits do not. The limits that the beta distribution imposes on the AUC closely track these previous approximations [12,13] and also match a predictive genomics simulation based on a multiplicative genetic model [10].

**Figure 3:**
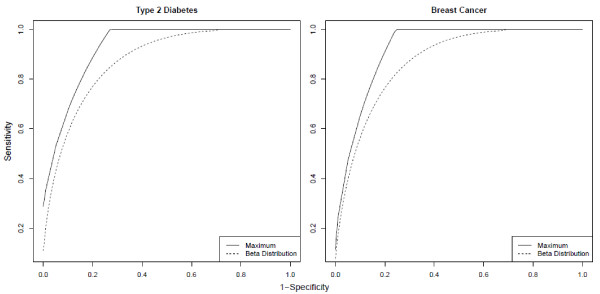
**ROC curves for type 2 diabetes and breast cancer from genomic profiles.** Maximal sensitivity / 1-specificity pairs for prediction of type 2 diabetes and breast cancer from full genomic profiles. The maximal pairs are compared to the pairs that would exist if the genetic risk distribution followed a beta distribution, which is consistent with previous reports [10,12,13].

Knowledge of this maximal limit on accuracy is beneficial in the case of type 2 diabetes (T2D), where early targeted intervention can be costly but effective [15]. Many prediction studies of T2D have been reported, yet the genetic contribution to their predictive accuracy has been disappointing: genes alone yield ~60% AUC, and adding genes to clinical risk factors yields incremental improvements of ~1-2% AUC [16,17]. The heritability of T2D *per se* (as opposed to related continuous traits with higher heritability, e.g. body mass index) was estimated to be 26% by a population-based twin study [18], with a prevalence of 13%. Applying our method to these statistics determines the maximum sensitivity/specificity pairs displayed in Figure 3, which show that, for example, if a specificity of 99% is desired, sensitivity cannot exceed 36%, and that if a sensitivity of 99% is desired, specificity cannot exceed 74%. Similarly, they determine the maximum achievable AUC for genetic prediction of lifetime T2D to be 89%. This motivates the search for additional genetic factors influencing risk for T2D.

Breast cancer has the same maximal AUC as T2D, albeit with a distinct ROC curve from T2D. Breast cancer was found to have a prevalence of 4% [19], and we calculated its heritability on the binary scale to be 11% (see Methods), which yields a maximum AUC of 89%. Although this is the same maximum AUC as for T2D, the sensitivity/specificity pairs for breast cancer (Figure 3) are not identical to those for T2D, owing to the different disease parameters. For example, to reach a specificity of 99%, sensitivity cannot exceed 24%, which is substantially lower than the corresponding maximal sensitivity of T2D when specificity is 99%. The divergence of these two ROC curves as specificity approaches 100% illustrates the importance of identifying the maximal ROC curve, rather than relying on the maximal AUC alone.

Heritability is the proportion of phenotypic variance explained by *all* genetic factors, but our analytic approach can treat the proportion of phenotypic variance explained by *any particular set* of factors. If the proportion of phenotypic variance explained by a particular set of genes is known, that proportion of variance explained could be substituted for heritability in our model. For instance, if a subset of genes could explain 50% of the genetic variance of T2D (i.e. explain 13% of phenotypic variance), then the maximum achievable AUC of this subset would be 80%.

Our method can also be applied in elucidating the maximum accuracy of predictors that integrate features such as gene expression, *de novo* mutation, body mass index, and lifestyle (which are not fully inherited). The proportion of variance explained by such an integrated predictor can then be greater than heritability. When there are no gene-environment interactions, this difference is the proportion of phenotypic variation that these features explain *independently* of genes. For example, weekly physical activity can explain 4% of phenotypic variance of T2D (see Methods), is moderately heritable [20], and was found to not interact with well-known gene variants in T2D [21]. Accordingly, the proportion of variance explained by the integrated predictor comprised of genomic profile and physical activity does not increment by the full 4% beyond the heritability of T2D. If the proportion of T2D variance that physical activity explains independently of genes was known to be only 3%, say, then the integrated predictor’s maximum AUC would be calculated based on a proportion of variance explained of 29% (sum of 26% and 3%), which yields a maximum AUC of 90%. If, however, we did not have an estimate for the proportion of T2D variance that physical activity explains independently of genes, then we could conservatively use 4% in the previous calculation, yielding a similar AUC. This analysis applies to predictors based on non-genetic features that are supplemented by genetics. In general, the estimation of the proportion of variance explained by integrated predictors is complicated by the interaction of genetic and non-genetic features; our method can nonetheless be applied when the interaction can be estimated or bounded. Note that genetic testing alone can still accurately predict outcome for some small, extreme risk groups (such as those with highly penetrant variants), but such a test will not benefit the general population without both a high sensitivity and specificity [22].

## Discussion

Our results are general and apply to any binary trait, and they rely on only two commonly estimated parameters. Although the quality of the results is only as good as the estimates of prevalence and heritability for the population in question, our method allows for ranges of prevalences and heritabilities to be considered, which can provide important insight into predictive accuracies. Nonetheless, care must be taken when applying these statistics, as different estimates apply in different situations. For example, in assessing limits to the prediction of lifelong risk, lifelong risk estimates should be used in place of prevalence estimates. In particular, the ballooning lifelong risk of T2D in the USA [23] implies genetic prediction of lifetime T2D will become more difficult.

The method that we present here can also be used to determine the potential benefit of a future genomewide association study (GWAS) in improving predictive accuracy. To do so, we refer to estimates of GWAS predictive power that were cleverly derived either by simulation studies [24] or closed-form considerations [25]. Both approaches measure the potential GWAS benefit in terms of the correlation of individuals’ genetic risk as predicted by the GWAS to their true genetic risk. We can use our results to connect this measure to AUC and sensitivity/specificity pairs by converting this correlation to a proportion of phenotypic variance explained. If *H*^2^ is the broad-sense heritability and *r* is the correlation of true to estimated genetic risk, then the proportion of phenotypic variance that the proposed GWAS may explain, *R*^2^, is given by [12]:

(2) $$R^{2}=r^{2}H^{2}$$

Using this approach, one may evaluate a proposed GWAS based on parameters such as sample size and the number of loci sampled.

Heritability estimates for any binary trait can be used by our method. Broad-sense heritability estimates are needed to cap predictive accuracy, since genetic predictors can exploit dominance and epistatic interactions not measured by narrow-sense heritability estimates. However, if a genetic predictor is constructed as an additive model in line with the assumptions of narrow-sense heritability, then its maximum accuracy can be calculated using narrow-sense heritability; thus, these estimates can also be used, albeit with a slightly different interpretation. Heritability estimates on the normal liability scale can be used after they are transformed to the observed binary scale, e.g. using the method proposed by Dempster and Lerner [8,9]. Heritability on the binary scale can be sensitive to prevalence [26], but its use avoids the assumption of normally-distributed liability, which requires that the trait be affected by many genes, all with small effect (normally-distributed liability effectively requires a purely unimodal genetic risk distribution). In fact, when variants with particularly large effects do exist—such as *APOE* in Alzheimer’s disease [27], *BRCA1* and *BRCA2* in breast and ovarian cancer [28], and *LRRK2* in Parkinson’s disease [29]—previous authors have suggested simulations in lieu of their analytical approximation [13]. Moreover, because liability cannot be measured, the distributional assumptions on liability are virtually untestable [30].

Our maximal ROC curves (Figure 3) can be substantially higher than those given by the beta distribution, which is an accurate proxy for multiple previous reports [10,12,13], indicating that the maximal accuracies of genetic prediction may be substantially higher than previously thought. This difference highlights the importance that the risk distribution can have in the power of genetic prediction. Furthermore, as we are only now beginning to uncover the risk distributions of common complex diseases, it seems important to understand the absolute, distribution-independent limits on genetic-test accuracy, which we present here.

## Conclusion

We have given exact limits on genetic prediction for any binary trait imposed by the epidemiological parameters of prevalence and heritability. Knowledge of these limits can help delineate the maximal benefits associated with genetic testing, which can allow for cost-benefit analyses, regulation, and clinical guidelines regarding genetic testing even before additional associations are identified. We have also illustrated how these limits can help us prioritize the allocation of research resources, by showing how they can assist in the prioritization and design of future association studies. The calculations presented in this paper could further be used to mitigate the possibility of investing in the development of a genetic test which could never be accurate enough to be of clinical relevance.

## Methods

To optimize over the set of risk distributions subject to the disease parameters of average risk and proportion of variance explained (PVE), we modeled a categorical distribution (which resembles a histogram) with *b + 1* bins located at *0, 1/b, 2/b, … , 1* representing risk groups, so *i/b* represents the conditional probability of disease given a set of factors for individuals in risk group *i* (e.g. people in the *1/b* group have risk *1/b*). An example of such a distribution is depicted in Figure 1. The probability that someone falls into bin *i* is *p*_*i*_, where the *p*_*i*_‘s (for *i = 0,…,b*) sum to one. We restrict the average risk (e.g. *prevalence*) and PVE (e.g. *heritability*) using two observations. (1) By the law of total probability, the unconditional probability of disease is simply the mean of the conditional risk distribution, i.e. it is equal to the average risk. (2) The PVE relates to the risk distribution through Equation 1. (Equation 1 can be understood as the R^2^ from the regression: *binary phenotype = risk + error*, where *risk* is a probability.)

Now, we perform a brief simplification of Equation 1. Following Wray et al. [24], we denote average risk by *k*, and for generality we work in terms of PVE instead of H^2^:

(3) $$PVE=1-\frac{\sum_{i}risk_{i}-\sum_{i}risk_{i}^{2}}{k(1-k)n}$$

(4) $$k(1-k)PVE=k(1-k)-\frac{\sum_{i}risk_{i}-\sum_{i}risk_{i}^{2}}{n}$$

(5) $$k(1-k)PVE=k(1-k)-k+\frac{\sum_{i}risk_{i}^{2}}{n}$$

(6) $$k(1-k)PVE+k^{2}=\frac{\sum_{i}risk_{i}^{2}}{n}$$

where *i* = *1,…,n* indexes individuals, *n* is the sample size, and *risk*_*i*_ is individual *i*’s genetic risk. We can relate the right-hand side of Equation 3 to risk groups as follows:

(7) $$\sum_{i=1}^{n}risk_{i}^{2}/n=\sum_{j=0}^{b}n_{j}risk_{j}^{2}/n={\sum_{j=0}^{b}p_{j}\left(\frac{j}{b}\right)}^{2}$$

Here, *n*_*j*_ individuals have risk *j/b*, i.e. they are assigned to risk group (histogram bin) *j*, and *p*_*j*_ *= n*_*j*_*/n* is the probability that a random individual is assigned to risk group *j*.

With this model of the risk distribution and constraints, we can identify the best-case AUC and optimal sensitivity/specificity pairs using the procedures detailed below. Because these procedures associate a single genetic risk distribution with the best-case AUC and a potentially different risk distribution with each optimal sensitivity/specificity pair, it is possible that only some of these sensitivity/specificity pairs may be realizable for a single trait in practice. Consequently, these sensitivity/specificity pairs cannot be used directly to derive the maximal AUC.

## Area under ROC curve

To model the AUC, we begin with the random variables *X* and *Y* whose probability density functions represent the risk distribution of those who will not and those who will get sick, respectively. These densities can be easily obtained through Bayes rule: $P\left(X=\frac{i}{b}\right)=\frac{\left(b-i\right)p_{i}}{b\left(1-k\right)}$ and $P\left(Y=\frac{i}{b}\right)=\frac{ip_{i}}{bk}$, where *k* is the average risk. Then, through its equality to the Mann–Whitney–Wilcoxon U statistic [31], the AUC is equal to P(X<Y)+P(X=Y)/2. We condition on *Y* to evaluate this expression:

(8) $$AUC=\sum_{i=1}^{b}P\left(Y=i/b\right)\left[\sum_{j=0}^{i-1}P\left(X=j/b\right)+\frac{P\left(X=i/b\right)}{2}\right]\text{.}$$

We would like to optimize this term, but unfortunately it is not convex, which would undermine our ability to identify the global optimum. However, after we substitute *p*_*0*_ with $1-\sum_{i=1}^{b}p_{i}$, our optimization of the AUC becomes a convex optimization problem:

(9) $$AUC=\frac{\sum_{i=1}^{b}ip_{i}\left[b-\sum_{j=1}^{b}bp_{j}+\sum_{l=1}^{i-1}\left(b-l\right)p_{l}+\left(b-i\right)p_{i}/2\right]}{b^{2}k\left(1-k\right)}$$

The numerator of this expression can be conveniently represented as *p*^*T*^*Qp + b*^*2*^*k*, where *Q* is a symmetric matrix whose entry at row *i* and column *j* is *-j(b + i)/2* for *i ≥ j*.

We then maximize this AUC over the vector *p* subject to the disease parameters of average risk (*k*) and proportion of variance explained (*PVE*):

(10) $$k=\sum_{i=1}^{b}\left(i/b\right)p_{i}$$

(11) $$k\left(1-k\right)PVE+k^{2}=\sum_{i=1}^{b}{\left(i/b\right)}^{2}p_{i}$$

where the sum of the *p*_*i*_‘s (for *i = 1,…,b*) must not exceed 1, and each *p*_*i*_ is bounded between 0 and 1.

The parameters *kPVE*, and *b* are predefined constants. Note that for *b = 100*, as well as for all the values of *b* we examined, *Q* is negative definite, so that this is a convex program. Hence, there are efficient solution methods to identify the global maximum. Using the *quadprog* package [32] in the R software [14], we solved this program for values of *k* and *PVE* with *b = 100*. When *b = 1000*, all maximal AUCs shown in Figure 2 change by less than 0.01%. In fact, using *b = 10* does not change any of these maximal AUCs more than 1% from that calculated with *b = 1000*. Note also that given an estimated risk distribution vector *p*, a researcher can directly calculate the AUC from the objective function. To calculate the AUC of the beta distribution for given levels of *k* and *PVE*, we discretized the beta distribution with parameters *a = k(1/PVE-1)* and *b = (1-k)(1/PVE-1)*, which uniquely satisfy the constraints.

## Sensitivity/specificity pairs

To represent each point on the optimal ROC curve, we model the best sensitivity (*Se*) and specificity (*Sp*) for any given risk threshold *t/b* in terms of the risk distribution. The logic is that the best a genetic test can do is identify true genetic risk, so it will declare those with a risk greater than the threshold as positive and those with a lower risk as negative. Mathematically, the sensitivity of the test is the probability of an individual testing positive for the trait (i.e. having risk of at least *t/b*) given that they are truly positive:

(12) $$\begin{array}{ll} \text{Se} & =\text{P}\left(\text{test}+\text{truly}+\right)=\text{P}\left(\text{test}+\&\text{truly}+\right)/\text{P}\left(\text{truly}+\right)\\ & =\left(\sum_{i=t}^{b}\frac{i}{b}p_{i}\right)/k=\frac{1}{bk}\sum_{i=t}^{b}ip_{i} \end{array}$$

Similarly, we can derive specificity:

(13) $$Sp=\frac{1}{b(1-k)}\sum_{i=0}^{t-1}(b-i)p_{i}$$

We optimized sensitivity for any given value of specificity, average risk, proportion of variance explained, and threshold using a linear programming model. This was implemented in the *lpSolve* package in R [14] using 1000 bins. We then optimized the sensitivities over the thresholds to obtain the maximal sensitivity for every specificity, average risk, and proportion of variance explained.

## Calculations for examples

To calculate the proportion of T2D variance explained by physical activity we used Equation 1, where the risk distribution was defined by the prevalence and the relative risks of exercise [33]. To calculate the heritability of breast cancer on the binary scale we used twice the difference in correlation between monozygotic and dizygotic twin pairs, where correlations were computed on binary outcomes from 44,788 pairs of Nordic twins [34].

## Abbreviations

ROC: receiver-operating characteristic; AUC: area under ROC curve; T2D: type 2 diabetes; GWAS: genomewide association study; PVE: proportion of variance explained.

## Competing interests

GMC has advisory roles in and research sponsorships from several companies involved in genome sequencing technology and personal genomics (see http://arep.med.harvard.edu/gmc/tech.html).

## Author’s contribution

JMD designed the study, carried out the analysis, and drafted the manuscript. DL designed the study and drafted the manuscript. JEG provided computing resources and helped direct the study. GMC helped direct the study. MFR designed the study and critically revised the manuscript. All authors read and approved the manuscript.

## Supplementary Material

Additional file 1 **Table of maximum AUCs.** These are the maximum AUCs corresponding to Figure 2 for all values of prevalence. Row names represent values of heritability (computed on the observed binary scale) or proportion of phenotypic variance explained, and column names represent values of prevalence. Click here for file

Additional file 2 **Table of maximum sensitivities for each specificity.** Rows represent the combination of heritability (H.sq, computed on the observed binary scale) and prevalence (Prev), while columns represent specificities. The elements are the maximal sensitivity in each case. Click here for file

Additional file 3 **Archive containing instructions *(readme.txt) *and computer code *(maxAcc.r) *to implement our algorithms.** The code is written in the free statistical language and environment R (http://www.r-project.org), relies on free R optimization packages, and is copyrighted by the permissive MIT license (http://www.opensource.org/licenses/mit-license.html). Updated versions are freely available for download at: http://code.google.com/p/max-accuracy-genetic-pred/. Click here for file
